# Sandblasting reduces dental implant failure rate but not marginal bone level loss: A systematic review and meta-analysis

**DOI:** 10.1371/journal.pone.0216428

**Published:** 2019-05-03

**Authors:** László Márk Czumbel, Beáta Kerémi, Noémi Gede, Alexandra Mikó, Barbara Tóth, Dezső Csupor, Andrea Szabó, Sándor Farkasdi, Gábor Gerber, Márta Balaskó, Erika Pétervári, Róbert Sepp, Péter Hegyi, Gábor Varga

**Affiliations:** 1 Department of Oral Biology, Faculty of Dentistry, Semmelweis University, Budapest, Hungary; 2 Institute for Translational Medicine, Medical School, University of Pécs, Pécs, Hungary; 3 Department of Pharmacognosy, Faculty of Pharmacy, University of Szeged, Szeged, Hungary; 4 Interdisciplinary Centre of Natural Products, University of Szeged, Szeged, Hungary; 5 Department of Public Health, Faculty of Medicine, University of Szeged, Szeged, Hungary; 6 Department of Anatomy, Histology and Embriology, Faculty of Medicine, Semmelweis University, Budapest, Hungary; 7 Second Department of Internal Medicine and Cardiology Centre, University of Szeged, Szeged, Hungary; University of Mississippi Medical Center, UNITED STATES

## Abstract

**Introduction:**

Sandblasting is one of the oldest implant surface modifications to enhance osseointegration. Regarding its superiority over machined surface controversies still exist. Our objective was to compare implant failures (IF) and marginal bone level (MBL) changes between sandblasted and machined dental implants by a meta-analysis utilizing the available data. The PROSPERO registration number of the meta-analysis is CRD42018084190.

**Methods:**

The systematic search was performed in Cochrane, Embase and Pubmed. Inclusion criteria included participants with neither systemic diseases, nor excessive alcohol consumption, nor heavy smoking. We calculated pooled Risk Ratio (RRs) with confidence intervals of 95% (CIs) for dichotomous outcomes (implant failure) and weighted mean difference (WMD) CIs of 95% for continuous outcomes (marginal bone level change). We applied the random effect model with DerSimonian-Laird estimation. I^2^ and chi^2^ tests were used to quantify statistical heterogeneity and gain probability-values, respectively.

**Results:**

Literature search revealed 130 records without duplicates. Out of these, seven studies met the inclusion criteria and all were included in data synthesis, involving 362 sand-blasted and 360 machined implants. The results indicate that there is an 80% (RR = 0.2 95% CI:0.06–0.67; I^2^ = 0.0% p = 0.986) lower among sandblasted compared to machined implants after one year of use and 74% (RR = 0.26 95% CI:0.09–0.74; I^2^ = 0.0% p = 0.968) five years of use, respectively. In contrast, there is no significant difference in MBL (WMD:-0.10mm, 95% CI:-0.20, 0.01; p>0.05; I^2^ = 0.0%, p = 0.560 and WMD:-0.01mm, 95% CI:-0.12, 0.09; p>0.05; I^2^ = 26.2%, p = 0.258) between the two implant surfaces after one and five years of use.

**Conclusions:**

This meta-analysis reveals that sandblasting is superior over machined surface in implant failure but not in marginal bone level in healthy subjects. It also points out the need for further randomized clinical trials with large sample size for objective determination of the clinical benefits of certain implant surface modifications.

## Introduction

Since machined titanium dental implants were first used [1], enormous effort has been put into research to enhance osseointegration and increase the life span of implants. Many parameters have been identified that influence the period of healing time and bone stability [2–4].

It has been suggested that surface roughness is one of the several key factors influencing the degree of biological integration and success rates of inserted implants [5–7]. As a result of extensive investigation, several surface modifications have emerged. These include sandblasting, acid-etching, anodization, plasma-spraying, coating with different bioactive surfaces and the combination of these [5, 8]. Generally, implant surface roughness is modified by these processes. For roughness classification, four categories exist: smooth (S_a_ < 0.5 μm), minimally rough (S_a_ = 0.5–1 μm), moderately rough (1 μm < S_a_ < 2 μm) and rough implant surfaces (S_a_ > 2 μm) [9].

Sandblasting was one of the first modifications invented, resulting in moderately rough or rough surfaces, and it is still used by many implant manufacturers [4, 7]. During the blasting process, ceramic particles such as titanium oxide, aluminum oxide or silica [10] are blustered onto the implant surface at high velocity [11]. The size of sand particles and their speed when they reach the implant surface are the key parameters influencing surface roughness [8, 12]. The size of the particles usually varies between 25–250 μm [8, 13]. As a result, the surface becomes irregular with depressions and pits, and roughness (S_a_) is between 1.2–2.2 μm [9, 14]. In contrast, machined surfaces are smoother, having only shallow grooves on the surface [8]. The roughness of a machined surface is usually between 0.5–1 μm [9].

Several *in vitro* studies have demonstrated the positive effects of sandblasted surfaces on osseointegration [7, 15, 16]. However, some preclinical and clinical investigations and reviews indicated that moderately rough surfaces may not perform better. These studies suggest that a rougher surface may modify the properties of biofilm formation and, therefore, bacteria could attach to the surface more easily [9, 16, 17]. Hence, the marginal bone around rough implants may be less stable [18] and more vulnerable to peri-implantitis [19, 20].

Although the attention and utilization shifted from machined to sandblasted surface, the scientific reason behind is not well-founded. In other words, for clinical practice, no clear and strong evidence exists to support the use of sandblasted implants over machined ones.

The RCTs investigating the effect of sandblasted implants applied relatively small sample sizes providing weak evidence. Conducting meta-analysis could overcome the weaknesses of the individual RCTs by increasing sample size and the validity of the statistical analysis Several review papers have been published on this topic[9, 20–22], which are, however, either not based on meta-analyses (because the authors, due to the great heterogeneity of the included studies, did not perform any) or even if they are, the meta-analyses performed combine all kinds of moderately rough surfaces. As an outstanding example, the most recent systematic review pooled together extremely heterogeneous studies, in which there were great differences in the study design. Thus, in addition to the results of RCTs, also those of uncontrolled trials and retrospective studies were combined in a single statistical analysis [20], thereby representing a very high level of bias. To our knowledge, no meta-analysis was performed involving exclusively RCTs, comparing the effect exerted on osseointegration by sandblasted implants with that exerted on it by machined implants. We assumed that identifying all relevant publications and conducting a meta-analysis might overcome the weaknesses of small sample size and increase the value of evidence in the topic.

The objective of the present meta-analysis and systematic review was to test the hypothesis that there are significant differences in implant failure rates and marginal bone level changes between sand-blasted and machined dental implants.

## Materials and methods

### Protocol and registration

This meta-analysis follows the PRISMA guideline [23]. The PRISMA checklist summarizing the content of this review is available in the supporting information (S1 Appendix).

The meta-analysis has been registered in Prospero (International Prospective Register of Systematic Reviews) database, 07/02/2018, registration number: CRD42018084190 (S2 Appendix).

### Eligibility criteria

The PICO (patient characteristics, type of intervention, control and outcome) format was applied to the following clinical question: are there significant differences concerning implant failure rates and marginal bone level loss between machined and sandblasted dental implants among healthy patients?

For analysis, we considered records published in scientific journals compiling with our selected PICO. Patient characteristics: edentulous or partially edentulous participants who do not have any systemic diseases that would affect the osseointegration of implants. Type of intervention: treating tooth loss with endosteal dental implants, having undergone sandblasting surface modification. Control: treating tooth loss with endosteal dental implants, with machined surface (no surface modification). Outcome: the number of implants survived at each check-up, and changes in marginal bone level around the implants, which are measured using radiographic images.

#### Inclusion and exclusion criteria

Publications meeting the following eligibility criteria were included: 1) randomized controlled trials; 2) intervention: sandblasted implants; 3) control group: machined implants; 4) healthy participants; 5) similar implant designs. Records written in English or available in English translations. Exclusion criteria: 1) any publication type other than randomized controlled trials; 2) application of growth factors; 3) bone augmentation; 4) surface modification only on the implant neck; 5) participants with systemic or local conditions affecting osseointegration; 6) gray or black literature.

### Information sources

A systematic search in English language limited to randomized controlled clinical trials was performed in three different major electronic databases (Cochrane Central Library, Embase and PubMed) with records published up to 20 August 2018. Besides electronic databases, an extensive hand search in the reference list of relevant articles and included records were also performed to find eligible records.

### Search

The following research string, was used in the Cochrane database: *“('machined'*:*ti*,*ab*,*kw or 'turned'*:*ti*,*ab*,*kw or 'blasted'*:*ti*,*ab*,*kw or 'sandblasted'*:*ti*,*ab*,*kw or 'sand-blasted'*:*ti*,*ab*,*kw) and ('dental'*:*ti*,*ab*,*kw or 'dentistry'*:*ti*,*ab*,*kw) and 'implant'*:*ti*,*ab*,*kw*" with Cochrane Library publication date to Aug 2018, in Trials.

The following search string was used for finding records in Embase: “*('machined'*:*ti*,*ab*,*kw OR 'turned'*:*ti*,*ab*,*kw OR 'blasted'*:*ti*,*ab*,*kw OR 'sandblasted'*:*ti*,*ab*,*kw OR 'sand-blasted'*:*ti*,*ab*,*kw) AND ('dental'*:*ti*,*ab*,*kw OR 'dentistry'*:*ti*,*ab*,*kw) AND 'implant'*:*ti*,*ab*,*kw AND 'controlled clinical trial'/de AND [english]/lim”*.

The following string was used to search on PubMed: *„(machined[Title/Abstract] OR turned[Title/Abstract] OR blasted[Title/Abstract] OR sandblasted[Title/Abstract] OR sand-blasted[Title/Abstract] OR sand blasted[Title/Abstract]) AND (dental[Title/Abstract] OR dentistry[Title/Abstract]) AND implant[Title/Abstract] AND (Clinical Trial[ptyp] AND ("0001/01/01"[PDAT]*: *"2018/08/20"[PDAT]) AND English[lang])”*

Besides electronic databases, the reference lists of relevant articles were also searched.

### Study selection

EndNote reference manger was used to organize and manage records. After removing duplicates, the remaining records were screened for suitability by two authors (L.M.Cz. and B.K.) based on the titles and abstracts of the published original papers. The eligibility of full texts of the remaining records was assessed by two reviewers independently (L.M.Cz. and B.K.). Disagreement between reviewers was resolved by discussion or, if it was necessary, by consulting with a third reviewer (G.V.).

### Data collection process and data items

Data extraction was performed by two authors independently (L.M.C. and K.B.) using a preconstructed standardized data extraction form. The following information was extracted: first author’s name, year of publication, sample size, population type (type of edentulism), average age of participants, gender distribution, design of the studies, implant system for intervention and control, outcome (implant failure rate, marginal bone loss), conclusion of each study. In case of disagreement, a third author (G.V.) was also involved.

### Risk of bias assessment

Quality and bias of the studies were evaluated according to the Cochrane Handbook [24], which is a broadly used guideline to assess randomized controlled trials. Studies were evaluated according to 8 domains. 1) Random sequence generation evaluates the strength of the method used for randomization. 2) Allocation concealment appraises the potential bias during allocation of the participants. 3) Blinding of participants and personnel assesses whether the patients and investigators were appropriately blinded to the treatment type. 4) Blinding of outcome assessment, radiographic outcome evaluates whether the personnel assessing x-ray images have been blinded. 5) Blinding of outcome assessment, clinical outcome appraises whether the clinical investigators evaluating the clinical outcome have been blinded. 6) Incomplete outcome data evaluate the risk of attrition bias due to withdrawals, loss of participants during follow ups and other missing data. 7) Selective reporting assesses whether all pre-determined outcomes have been measured and reported. 8) Other bias evaluates any other type of bias not falling into the previous 7 domains [24].

### Summary measures and synthesis of results

Pooled Risk Ratio (RRs) with 95% confidence intervals (CIs) and weighted mean difference (WMD) with 95% CIs were calculated for dichotomous outcomes (IF) and for continuous outcomes (MBL change expressed in mm) respectively. Negative values in MBL change indicate a decrease in marginal bone level. Negative values of weighted mean differences indicate a greater decrease in MBL in sand-blasted implants compared to machined ones. Criteria for implant failure were defined according to Albrektsson et al. [25] Implant number was chosen as statistical unit. We only considered results credible if raw data for meta-analysis could be drawn from at least three records. We applied the random effect model with DerSimonian-Laird estimation. I^2^ and chi-square tests were used to quantify statistical heterogeneity and gain probability-values, respectively; p<0.1 indicated a significant heterogeneity. [24] All statistical analyses were performed using STATA 15.0.

### Risk of bias across studies and additional analyses

Sensitivity analysis was performed by omitting studies (one by one) from the analyses and recalculating them in order to investigate the impact of the individual studies on the summary estimate. To check for publication bias, a visual inspection of funnel plots was performed.

## Results

### Study selection

**Fig 1:**
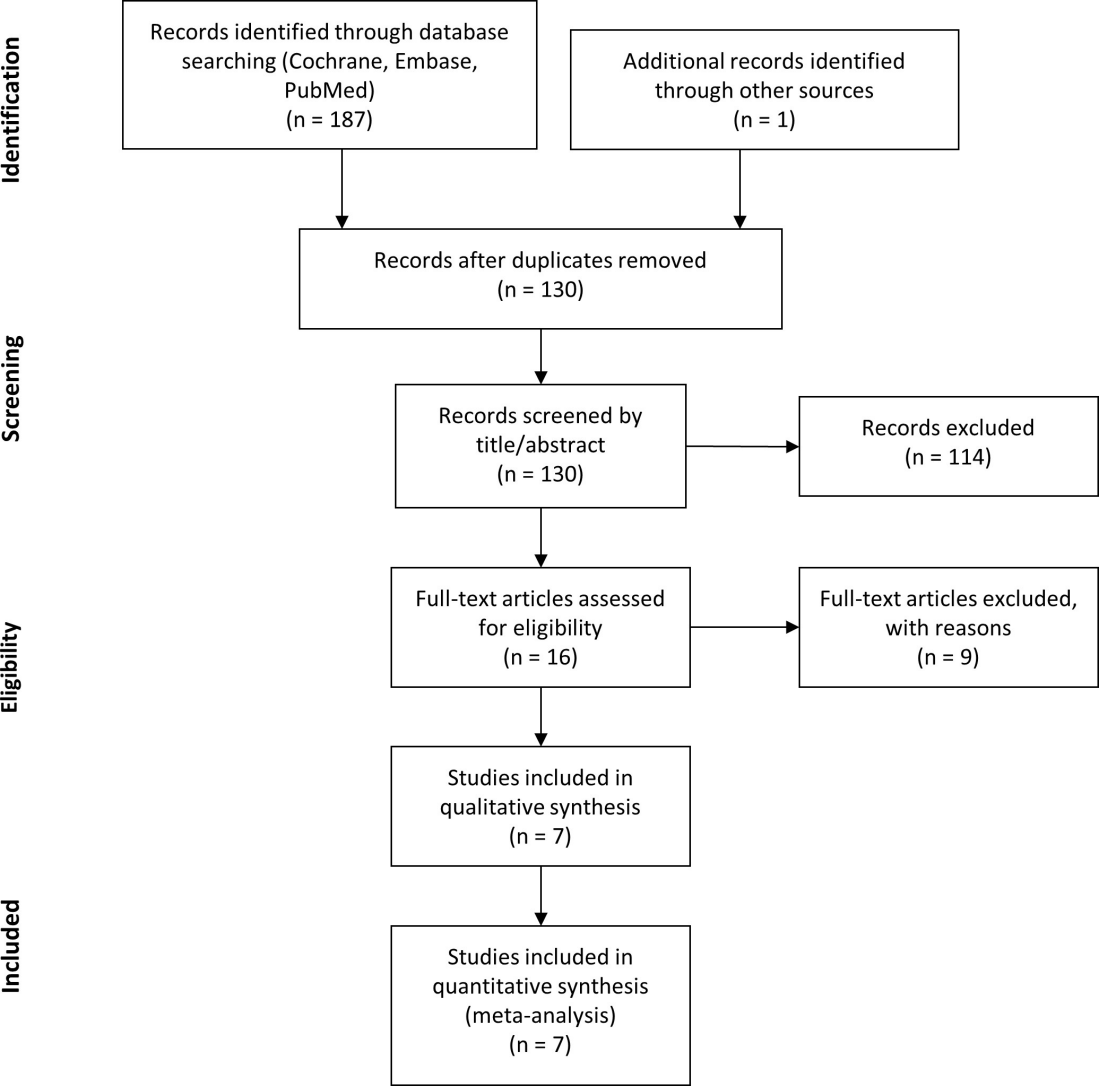
PRISMA flow diagram of study selection process.

During the study selection process, a total of 188 records were identified, including one record found in the reference list of related articles. After removing duplicates, 130 items remained. During the screening process, 114 records were excluded due to reasons such as other surface modification (n = 38) or different objectives (n = 76), investigating populations with systemic disease, evaluating surgical protocols, or comparing different macro designs of implants. For full-text evaluation 16 records were searched. Out of these publications nine records were excluded. The reasons for exclusion are explained below. Seven studies were eligible for qualitative and quantitative analysis [19, 26–31] (Fig 1).

### Study characteristics

#### Description of excluded studies

Out of the nine excluded records, three records were not eligible because of evaluating other surface modifications than the ones investigated in this meta-analysis [32–34]. Two studies reported on previous results of ongoing studies that have been republished in updated records [35, 36]. Two other records investigated different populations (periodontitis-susceptible) [37, 38]. One record was not RCT [39] and one other paper did not describe the surface modification used [40].

#### Description of the included studies

All involved studies were randomized controlled trials. A total of 722 implants (362 sandblasted and 360 machined) were included in the data synthesis. The populations represented in these studies were uniform, patients with alcohol and drug consumption or other medication abuse were excluded. Exclusion criteria also included bruxism, uncontrolled diabetes mellitus or any other significant medical condition that would affect the process of osseointegration. The mean age of participants in the studies varied between 50 and 58 years. In the five studies four different implant systems (Astra Tech, Brånemark, Steri-Oss and Southern Implants) were used. All implants in the control group had minimally rough surface and all implants in the intervention group had moderately rough surface [9].

All study groups except one [26] followed the two-stage protocol [41]. However, even in the study using one-stage protocol, implants were only loaded 3 months following healing at the lower jaw, and 6 months of healing at the upper jaw. Out of the five studies two [19, 26] treated edentulism with overdentures, another two [27, 30] used fixed partial bridges. One study [42] achieved rehabilitation with full arch bridges. The shortest follow-up was 2 years long, and the longest lasted for 16 years [29]. Each study provided information on implant failure rate and marginal bone level change calculated from blinded radiographic measurements. In one study [26], only two out of four groups were included, two groups using immediate loading protocol were not included in the analysis. Additionally, MBL measurements of Astrand et al. [31] and Ravald et al. [28]were excluded since the reported patient-based data could not be converted to implant-based data to match the statistics of other studies. A detailed description of these studies is shown in Tables 1 and 2.

**Table 1:** Summary of study characteristics.

Author	Åstrand et al. (2004)andRavald et al. (2013)*	Gotfredsen et al. (2001)	Steenberghe et al. (2000) and Jacobs et al. (2010)*	Tawse-Smith et al. (2002)	Vroom et al. (2009)
**Study type**	block randomization separate for upper and lower jaw, with equal probability of receiving either implant type	alternating implant placement	split-mouth design	random allocation to either implant system on a one-by-one basis	alternating implant placement
**Country**	Sweden	4 Scandinavian countries	Belgium	New Zealand	not stated
**Age**	$\bar{x}$ = 61.5	$\bar{x}$ = 53	$\bar{x}$ = 59.7	55–80	$\bar{x}$ = 53
**Number of participants**	males: 28, females: 38	males: 25, females: 25	males: 6, females: 12	total: 48	males: 7, females: 13
**Extent of teeth loss**	edentulous	partially edentulous	partially edentulous	edentulous (mandible only)	edentulous (mandible only)
**Sand-blasted implant (intervention)**	Astra Tech implants	Astra Tech implants	Astra Tech implants	Southern Implants	Astra Tech implants
**Machined implants (control)**	Branemark System MK II	Astra Tech implants	Branemark System MK II	Sterioss	Astra Tech implants
**Surgical protocol**	two-stage technique (3 months and 6 months healing in the lower and upper jaw respectively before abutment placement	two-stage technique (3–4 months and 6–7 months healing in the lower and upper jaw respectively before abutment placement	two-stage technique (3–4 months and 6–7 months healing in the lower and upper jaw respectively before abutment placement	one-stage technique (3 months of healing before loading)	two-stage technique (3–4 months healing before abutment placement

*The publications of Ravald et al (2013) and Jacobs et al (2010) are the continuations of the studies published by Åstrand et al (2004); and Steenberghe et al (2000) respectively.

**Table 2:** Summary of study characteristics.

Author	Åstrand et al. (2004) and Ravald et al. (2013)*	Gotfredsen et al. (2001)	Steenberghe et al. (2000) and Jacobs et al. (2010)*	Tawse-Smith et al. (2002)	Vroom et al. (2009)
**Type of prosthesis**	full-arch fixed bridges	screw retained fixed partial prosthesis	screw retained fixed partial prosthesis	implant supported overdenture	implant supported overdenture
**Outcome**	IF, MBL change, BOP, plaque accumulation, pain, suprastructure complications	IF, MBL change, BOP, paraesthesia, periimplant inflammation, pain, suprastructure complications	IF, MBL change, sulcus bleeding index, PPD presence of plaque	IF, MBL change, sulcus bleeding index, PPD, implant stability measurement (Periotest), modified plaque index	IF, MBL change, bleeding index. PPD, presence of calculus
**Follow-up time**	5 and 12*years	5 years	2 and 15*years	2 years	12 years

*The publications of Ravald et al (2013) and Jacobs et al (2010) are the continuations of the studies published by Åstrand et al (2004); and Steenberghe et al (2000) respectively.

IF: implant failure

MBL: marginal bone level

BOP: bleeding on probing

PPD: probing pocket depth

### Risk of bias within studies

Bias in the studies was assessed according to the Cochrane Risk of Bias Tool. All seven included studies were included in the risk of bias assessment, however two pairs of studies [28, 31] and [27, 29] were evaluated together because the study of Ravald et al [28] and Jacobs et al. [29] are the continuation of previous studies of Åstrand and coworkers [31] and those of Steenberghe and coinvestigators [27], respectively.

**Fig 2:**
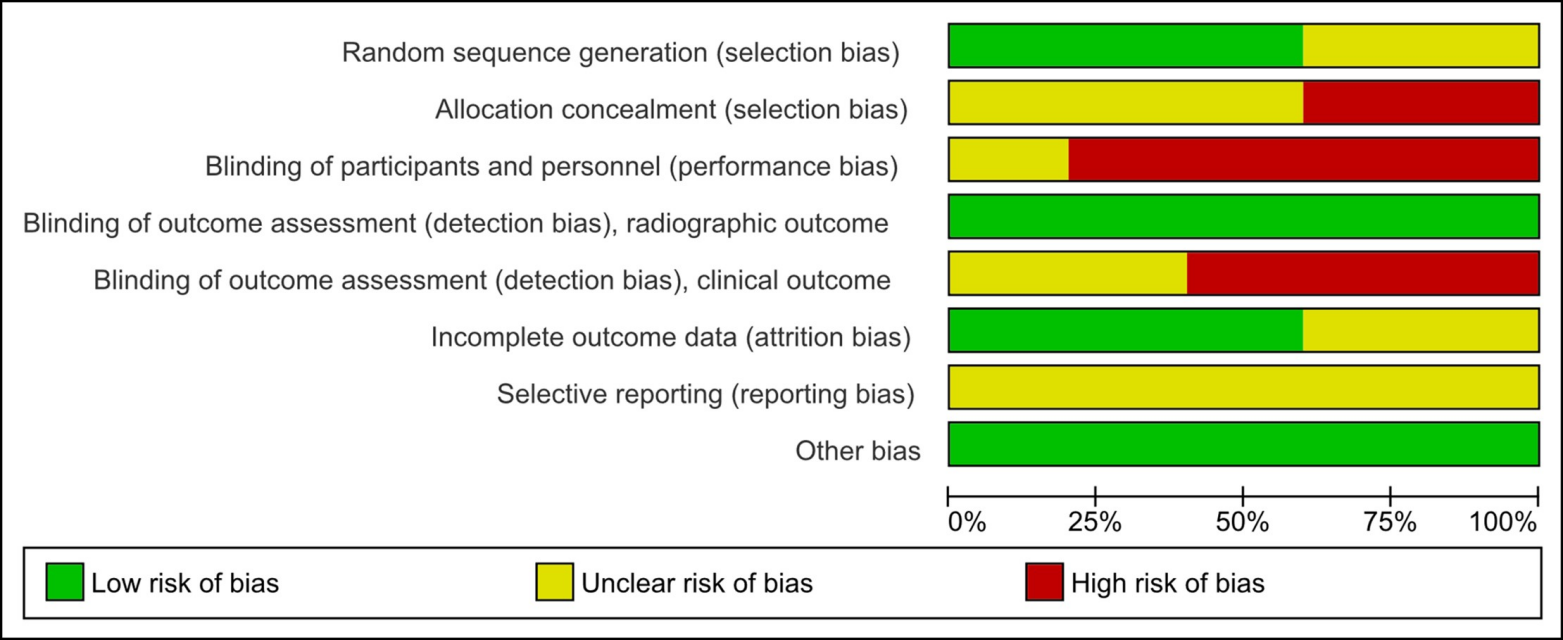
Risk of bias graph. Percentage of each risk of bias item across included studies.

Two studies [26, 27] had unclear random sequence generation, and other two [19, 30] had a high risk of allocation concealment, due to the predictable sequence generation process used. All studies performed blinding during the evaluation of x-ray images. However, due to its nature, no blinding could be carried out evaluating the implants clinically. Dropouts were identified in four studies [19, 26, 30, 31], two of these with unclear risk of bias.[19, 26]. Access was not gained to study protocols or trial registers, however, no intext evidence of selective reporting was found. Fig 2, S1 Table and S3 Appendix contain the summary of the risk of bias assessment.

### Results of individual studies and synthesis of results

#### Sandblasted implants are better than machined implants concerning implant failure at 1, 2 and 5–6 years

**Fig 3:**
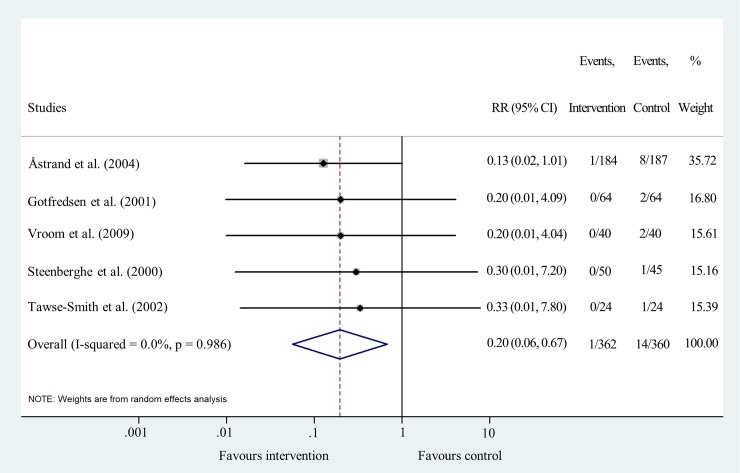
Forest plot analysis of implant failure rate after one year.

Data for implant failure analysis after one year were pooled from five studies [19, 26, 27, 30, 31]. The results show that there is an 80% lower risk for sand-blasted implants to fail compared to machined implants after one year of use (RR = 0.20 95% CI: 0.06–0.67; I^2^ = 0.0% p = 0.986) (Fig 3 and S1 File).

**Fig 4:**
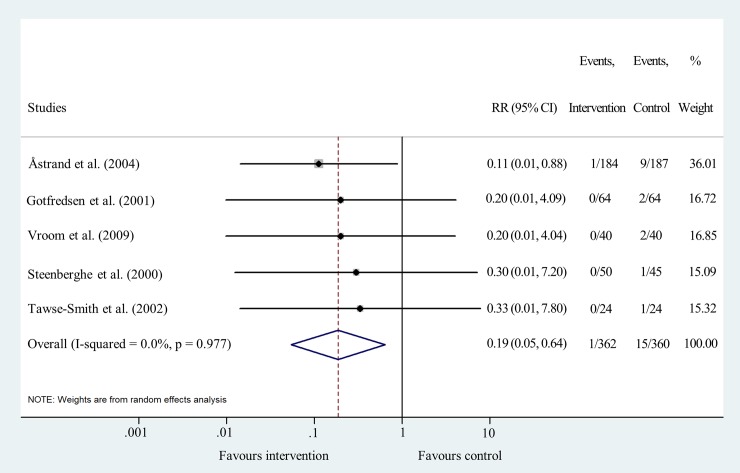
Forest plot analysis of cumulative implant failure rate after two years.

Data for cumulative implant failure after two years could be pooled from five studies [19, 26, 27, 30, 31]. The meta-analysis revealed that the risk of sand-blasted implant failure is 81% lower than that of machined implants (RR = 0.19 95% CI: 0.05–0.64; I^2^ = 0.0% p = 0.977) (Fig 4 and S1 File).

**Fig 5:**
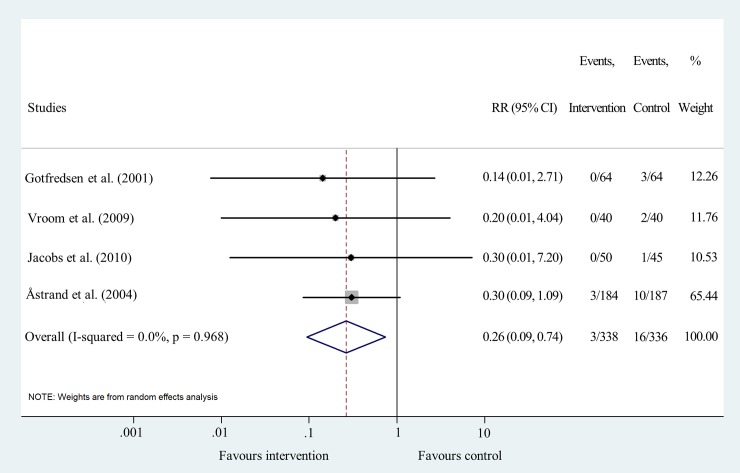
Forest plot analysis of cumulative implant failure rate after 5/6 years.

Data for analyzing the effect of sandblasting on implant failure after five or six years’ follow-up were pooled from four studies [19, 29–31]. The results indicate that there is a 74% lower risk of sandblasted implants to fail (RR = 0.26 95% CI: 0.09–0.74; I^2^ = 0.0% p = 0.968) (Fig 5 and S1 File).

**Fig 6:**
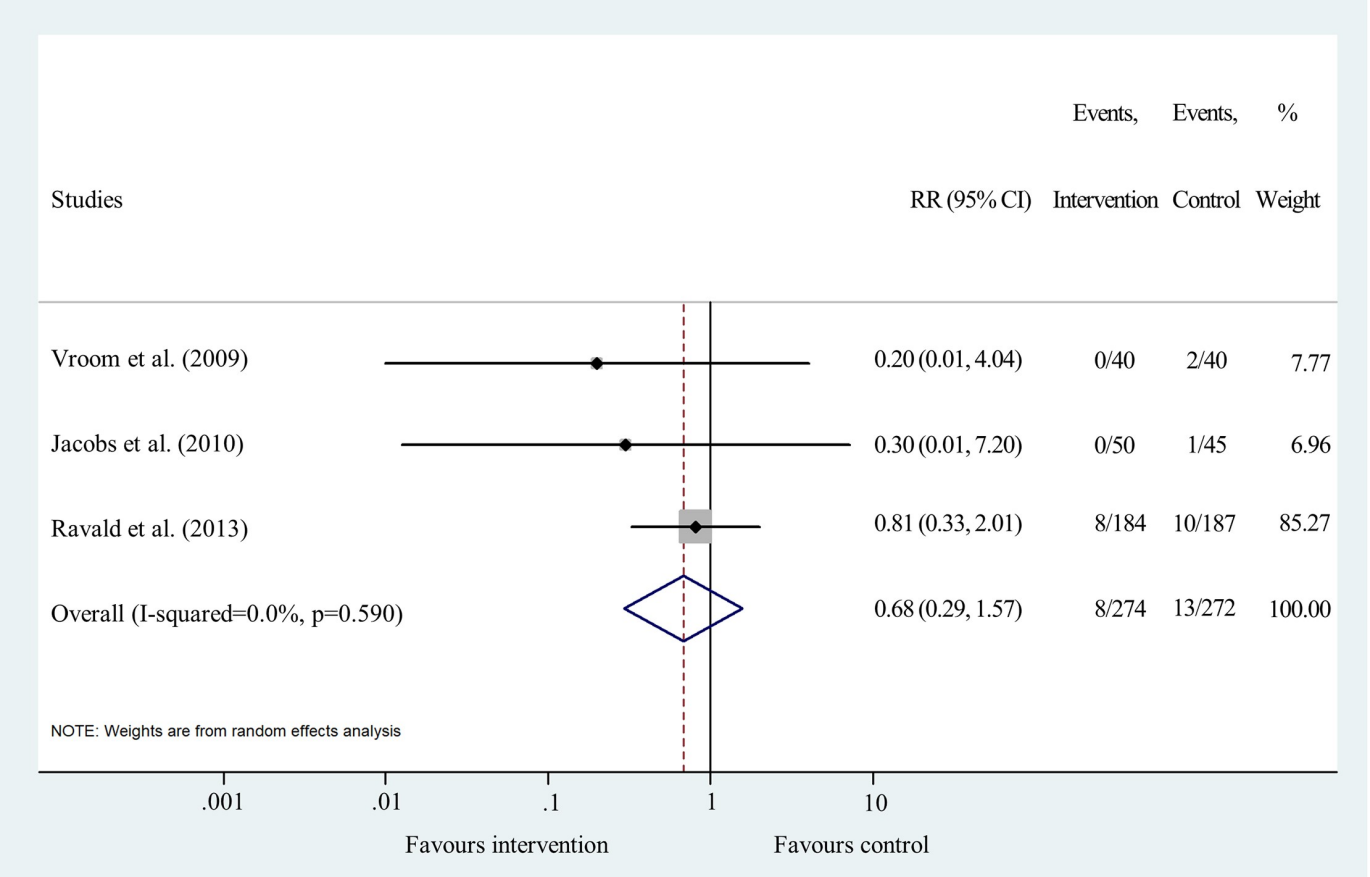
Forest plot analysis of cumulative implant failure rate after 12/15 years.

Results for cumulative IF after 12–15 years were synthesized from 3 studies [19, 28, 29]. Results show that there is no significant difference between the two treatment types (RR = 0.68 95% CI: 0.29–1.57; I^2^ = 0.0% p = 0.590) (Fig 6 and S1 File).

### No detectable difference in MBL between sand-blasted and machined implants after 5 years of follow up

**Fig 7:**
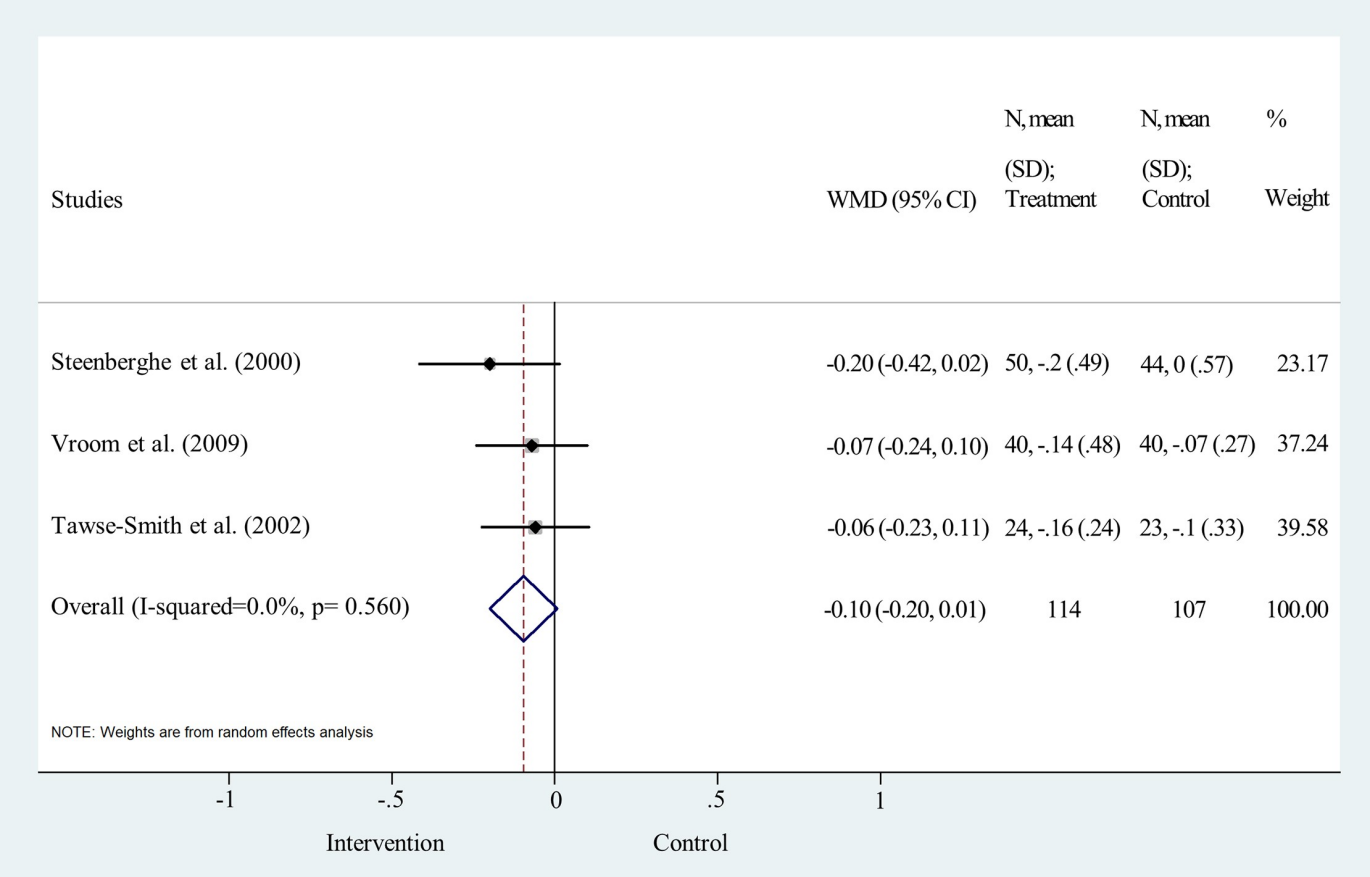
Forest plot analysis of marginal bone level change after one year.

**Fig 8:**
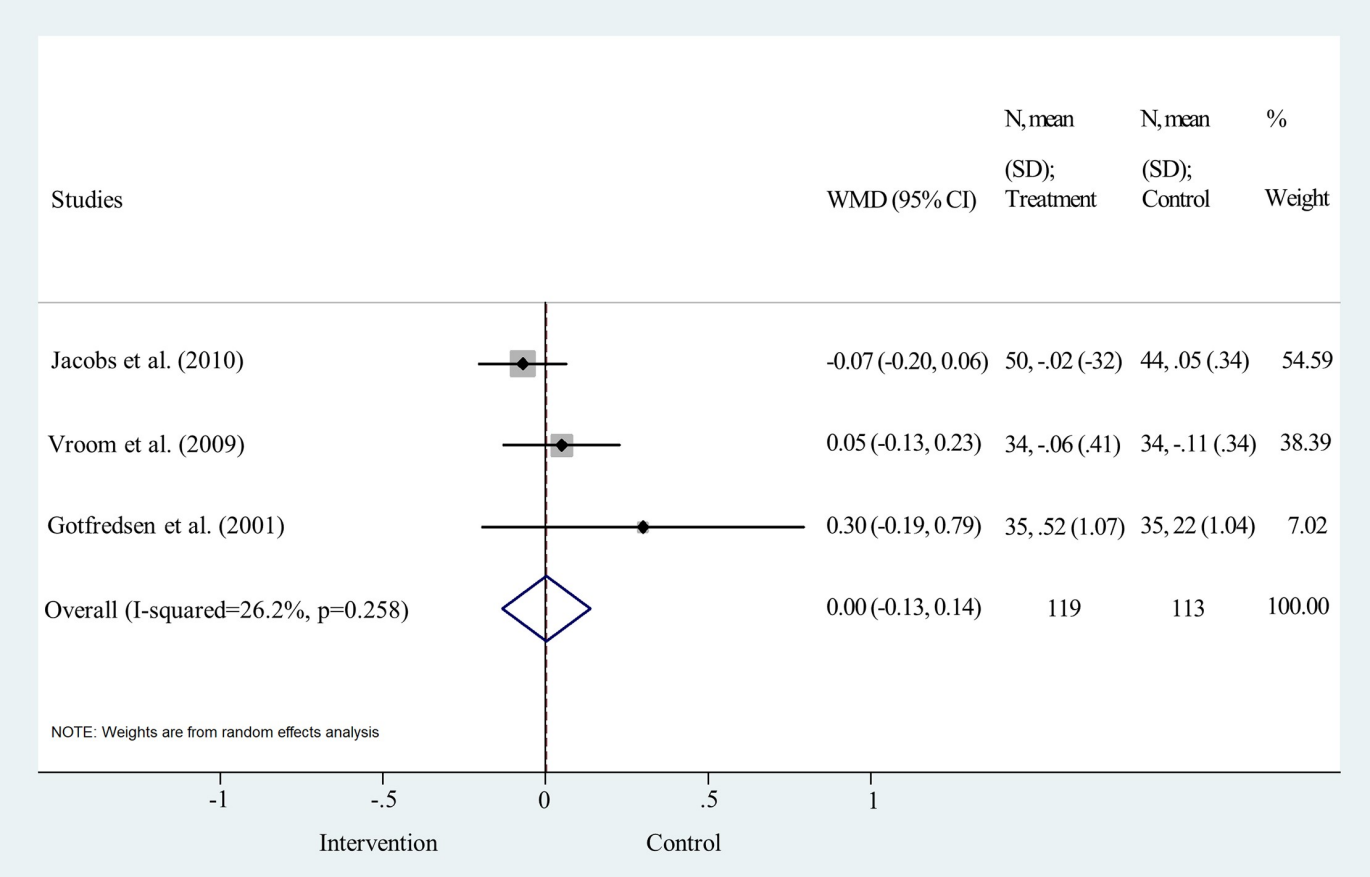
Forest plot analysis of marginal bone level change after 5 years.

MBL change was analyzed one and five years after the delivery of the final prosthesis One-year data were pooled from three studies [19, 26, 27]. No significant difference was found between the two surface treatments, (weighted mean difference = -0.10, 95% CI: -0.20–0.01; p>0.05; I^2^ = 0.0%, p = 0.560) (Fig 7 and S1 File). Data for 5-year analysis were pooled from three studies [19, 29, 30]. The statistical analysis clearly shows that the difference is not significant between the two implant surface types, the line of null effect falls within the range of the confidence interval (weighted mean difference = 0.00, 95% CI: -0.13–0.14; p>0.05; I^2^ = 26.2%, p = 0.258) (Fig 8 and S1 File).

### Risk of bias across studies and additional analysis

Funnel plot analyses indicated a moderate level of publication bias (S4 Appendix). Statistical heterogeneity was not important in the results of IF at all time points. I^2^ values were 0.0% and p values varied between 0.590 and 0.986 (Figs 3, 4, 5 and 6). Heterogeneity was also negligible in the results of MBL change at 1 year (I^2^ = 0.0%, p = 0.560) (Fig 7). I^2^ (26.2%) and p (0.258) values indicated a slightly higher level of statistical heterogeneity for the results of MBL change at 5 years (Fig 8), however, this was still considered insignificant. [24].

Sensitivity analysis showed that the removal of the study of Astrand et al. [31] decreases the significance of the results of pooled risk ratio analysis at one, two and five/six years following implantation. This is most likely due to the large sample size of that study compared to the other RCTs.

## Discussion

### Summary of evidence

As a result of extensive investigations conducted in the past decades, several methods for implant surface modifications have emerged and numerous studies claimed superiority for one or other roughened surfaces. [43–45] However, no evidence supports a single decisive hypothesis. Therefore, the contradictory conclusions of the literature require further studies and careful re-analysis. As our objective stated, we re-evaluated the performance of sand-blasted implant surface over machined ones. To obtain the highest level of evidence, a meta-analysis was conducted including only RCTs available on the topic but excluding uncontrolled trials and retrospective studies. Implant stability was evaluated by measuring MBL changes and comparing cumulative implant failure rates. After appropriate selection, seven RCTs, 202 patients, having 362 sand-blasted and 360 machined implants could be included in our complex approach.

Our meta-analysis revealed that implant failure rates were significantly different between machined implants and sand-blasted ones. In contrast, the results of the individual RCTs could not reveal a significant difference between the two types of surfaces. This gained difference reflects the increased number of samples and the high power of statistical methods of meta-analysis. Most implant failures happened during the first year after implantation. The reason for this could be that the surface modification of sandblasted implants creates a rougher surface which enhances the processes of bone formation on the implant itself. [46] Independent researchers published similar observations on other moderately rough surfaces, too. [47] Our results also show that after one year, when osseointegration has already taken place, the difference between the two surfaces diminish and the significance of the difference between implant failures disappear. Additionally, a histological study, using small sample size, also confirmed that, in the long term, both implant types maintained a decent level of osseointegration. It was found that after 5 years the bone-implant contact level was 92.7% for machined and 81.2% for sand-blasted implants. [48].

In the case of MBL change, our meta-analysis demonstrated no significant difference between the two implant types. The results of the individual RCTs included in our meta-analysis did not reveal any significant difference either. In contrast, a recent meta-analysis and also a novel review comparing machined implants to surface-modified implants concluded that rough implants may cause more bone loss [9, 20]. The discrepancy between the meta-analysis reported by Doornewaard and coworkers [9] and our results could arise from the fact that we only examined sandblasted implants and excluded all other surface modifications in order to decrease heterogeneity. Additionally, they might have included patients with periodontitis, which was an exclusion factor in our case. Moreover, Wennerberg et al. [20] included not only RCTs, but also uncontrolled trials and retrospective studies in their analysis. Thus, they used a different statistical approach yielding high statistical heterogeneity, which might fundamentally influence the outcome [20]. Nevertheless, in all included studies, MBL measurements resulted in high standard deviations, which hinder any accurate statistical comparison. Therefore, the results have to be interpreted carefully.

However, some general trends are indicated by literature data. Åstrand and coworkers argued that the greatest loss in marginal bone occurred between implant placement and prosthesis connection [31]. This change was found to be greater in machined than in sand-blasted implants [31]. Unfortunately, no published data on MBL change between implant placement and prosthesis connection are available for meta-analysis. Furthermore, based on data shown in Fig 7, the mean bone change within the RCTs was between 0 and -0.10 mm for machined, -0.14 and -0.2 mm for sand-blasted implants from baseline to the first year, which is, in fact, very small [26, 31]. According to Ravald and coinvestigators, the mean annual bone loss decreased gradually after five years. The annual mean bone attachment change was -0.02 and -0.04 mm for machined and sand-blasted implants, respectively, between five years and the end of the 12–15 years’ follow-up period [28]. In addition, there is evidence that bone gain can also occur around implants. The RCT of Åstrand et al. reported more than 0.6 mm increase in MBL around 4 sandblasted and 2 machined implants over five years [31]. Vroom and coworkers also noted an increase in MBL around some implants with not much difference between the two surface types. The authors of this study argue that bone gain is a result of increased bone corticalization [19].

The different trends in MBL change and IF concerning the two implant types can be explained by the differences between the two measuring methods. MBL measurements can only detect bone changes when the implant is still stable at the annual checkup. If bone resorption takes place so quickly that all the bone is resorbed within a year, the implants will be labeled to have failed and excluded from the MBL measurements, hence they no longer influence MBL changes. Indeed, numerous studies show that the initial bone formation takes place at a faster rate around rough surface implants than around machined ones [7, 49, 50]. This may also explain why fewer sand-blasted implants failed compared to machined ones in the first year after implantation. In addition, fast healing remains a key attribute of rough implants since healing time is a key element in modern implantology and implants with faster healing are prioritized [51, 52].

The present study has a clear message for clinicians. We hypothesized that, concerning implant failure rates and marginal bone level loss, there are significant differences between sandblasted and machined dental implants. Our meta-analysis provided evidence that sandblasting, indeed, significantly lowers implant failure rates although does not significantly affect marginal bone level changes. Thus, we recommend the use of sandblasted, moderately rough implants for patients with no systemic diseases as such implants support the osseointegration process with fewer complications than machined implants.

### Limitations

A major limitation of the present paper is the relatively small number of randomized controlled trials available regarding this topic. Despite the large number of records found by the systematic search, only seven could be included. The limited number of reported data makes it impossible to perform sub-group analyses and to thoroughly investigate the causes behind certain trends. Another issue that hinders in-depth analysis is the inhomogeneous reporting of outcome parameters. Some studies report the data separately for the lower and upper jaws, whereas others only report combined data. All studies reported one or two clinical parameters such as bleeding on probing. However, the use of different reporting schemes made comparison impossible for bleeding on probing tests, among others. Another limitation of the present work is that its conclusions apply only to healthy populations. There are several confounding factors which might create unfavorable conditions for moderately rough implants, such as patients with severe periodontitis [53]. However, these conditions were excluded from our analysis. Uncontrolled or unknown confounding factors not evenly affecting intervention and control groups may also contribute to differences in the outcomes. Finally, limitations of this meta-analysis include the heterogeneity of the implants used. Although implants with identical macro designs would be preferred, this was not really possible.

In conclusion, within the limitations of this meta-analysis, the results reveal that sandblasting is superior over machined surface concerning implant failure. On the other hand, no significant difference was found regarding marginal bone level changes between the two implant types. Our in-depth analysis of the literature also highlights that results are highly sensitive to heterogeneity and study design, which may lead to contradictory conclusions. In the future, consistent reporting on more clinical outcomes such as bleeding on probing, pocket probing depth and implant success rates are needed. Evaluation could be more meaningful if implant success is evaluated by RCTs rather than case-based implant failure studies. Therefore, a comprehensive protocol should be compiled to guide clinicians conducting valuable RCTs evaluating implant performance in order to decrease heterogeneity of papers and to increase clinical applicability.

## Supporting information

S1 Appendix PRISMA checklist. (PDF)

S2 Appendix PROSPERO registration. (PDF)

S3 Appendix Risk of bias summary: Review authors' judgements about each risk of bias item for each included study. (TIFF)

S4 Appendix Publication bias (funnel plots). (PDF)

S1 Table Detailed evaluation of risk of bias of individual studies. (PDF)

S1 File Data included in meta-analysis. (XLSX)
