# Supplementary material for: Sandblasting reduces dental implant failure rate but not marginal bone level loss: A systematic review and meta-analysis
Source: PLoS One. 2019 May 3;14(5):e0216428. doi: 10.1371/journal.pone.0216428 (PMC6499471; doi:10.1371/journal.pone.0216428)
Supplement: S2 Appendix — (PDF) [file pone.0216428.s002.pdf]

# Systematic review

## 1. * Review title.

Give the working title of the review, for example the one used for obtaining funding. Ideally the title should state succinctly the interventions or exposures being reviewed and the associated health or social problems. Where appropriate, the title should use the PI(E)COS structure to contain information on the Participants, Intervention (or Exposure) and Comparison groups, the Outcomes to be measured and Study designs to be included.

Survival Rates and Marginal Bone Level Changes of Sand-Blasted versus Machined Dental Implants: Meta-Analysis

## 2. Original language title.

For reviews in languages other than English, this field should be used to enter the title in the language of the review. This will be displayed together with the English language title.

## 3. * Anticipated or actual start date.

Give the date when the systematic review commenced, or is expected to commence.

25/09/2017

## 4. * Anticipated completion date.

Give the date by which the review is expected to be completed.

14/01/2019

## 5. * Stage of review at time of this submission.

Indicate the stage of progress of the review by ticking the relevant Started and Completed boxes. Additional information may be added in the free text box provided.
Please note: Reviews that have progressed beyond the point of completing data extraction at the time of initial registration are not eligible for inclusion in PROSPERO. Should evidence of incorrect status and/or completion date being supplied at the time of submission come to light, the content of the PROSPERO record will be removed leaving only the title and named contact details and a statement that inaccuracies in the stage of the review date had been identified.
This field should be updated when any amendments are made to a published record and on completion and publication of the review.

The review has not yet started: No

| Review stage | Started | Completed |
|---|---|---|
| Preliminary searches | Yes | Yes |
| Piloting of the study selection process | Yes | Yes |
| Formal screening of search results against eligibility criteria | Yes | No |
| Data extraction | No | No |
| Risk of bias (quality) assessment | No | No |
| Data analysis | No | No |

Provide any other relevant information about the stage of the review here (e.g. Funded proposal, protocol not yet finalised).

## 6. * Named contact.

The named contact acts as the guarantor for the accuracy of the information presented in the register record.

László Márk Czumbel

## Email salutation (e.g. "Dr Smith" or "Joanne") for correspondence:

Dr Czumbel

## 7. * Named contact email.

Give the electronic mail address of the named contact.

czumbel.laszlo@dent.semmelweis-univ.hu

## 8. Named contact address

Give the full postal address for the named contact.

## 9. Named contact phone number.

Give the telephone number for the named contact, including international dialling code.

## 10. * Organisational affiliation of the review.

Full title of the organisational affiliations for this review and website address if available. This field may be completed as 'None' if the review is not affiliated to any organisation.

Department of Oral Biology, Semmelweis University, Budapest
Institute for Translational Medicine, Medical School, University of Pécs

## Organisation web address:

## 11. Review team members and their organisational affiliations.

Give the title, first name, last name and the organisational affiliations of each member of the review team. Affiliation refers to groups or organisations to which review team members belong.

## 12. * Funding sources/sponsors.

Give details of the individuals, organizations, groups or other legal entities who take responsibility for initiating, managing, sponsoring and/or financing the review. Include any unique identification numbers assigned to the review by the individuals or bodies listed.

EFOP GRANT NUMBER: EFOP-3.6.2.-16-2017-00006

## 13. * Conflicts of interest.

List any conditions that could lead to actual or perceived undue influence on judgements concerning the main topic investigated in the review.

None

## 14. Collaborators.

Give the name and affiliation of any individuals or organisations who are working on the review but who are not listed as review team members.

## 15. * Review question.

State the question(s) to be addressed by the review, clearly and precisely. Review questions may be specific or broad. It may be appropriate to break very broad questions down into a series of related more specific questions. Questions may be framed or refined using PI(E)COS where relevant.

Is there a significant difference in implant survival and marginal bone loss between sand-blasted and machined dental implants?

## 16. * Searches.

Give details of the sources to be searched, search dates (from and to), and any restrictions (e.g. language or publication period). The full search strategy is not required, but may be supplied as a link or attachment.

We will perform our search in three electronic databases: Cochrane Library, EMBASE and PubMed. In addition, reference lists of eligible studies and review articles will be also searched.
The search query for EMBASE is (('machined':ti,ab,kw OR 'turned':ti,ab,kw OR 'blasted':ti,ab,kw OR 'sandblasted':ti,ab,kw OR 'sand-blasted':ti,ab,kw) AND 'dental':ti,ab,kw OR 'dentistry':ti,ab,kw) AND 'implant':ti,ab,kw AND [randomized controlled trial]/lim. Similar search terms are used for the other two databases too. Eligibility for inclusion will be decided following the inclusion/exclusion criteria.

## 17. URL to search strategy.

Give a link to the search strategy or an example of a search strategy for a specific database if available (including the keywords that will be used in the search strategies).

Alternatively, upload your search strategy to CRD in pdf format. Please note that by doing so you are consenting to the file being made publicly accessible.

Do not make this file publicly available until the review is complete

## 18. * Condition or domain being studied.

Give a short description of the disease, condition or healthcare domain being studied. This could include health and wellbeing outcomes.

Treatment of teeth loss with dental implants.

## 19. * Participants/population.

Give summary criteria for the participants or populations being studied by the review. The preferred format includes details of both inclusion and exclusion criteria.

Inclusion criteria: 1) randomized controlled trials; 2) control group: machined implants; 3)intervention: sand-blasted implants; 4) healthy participants, 5) similar implant design. Exclusion criteria: 1) using growth factors; 2) bone augmentation; 3) surface modification only on the implant neck; 4) participants with systemic condition affecting osseointegration.

## 20. * Intervention(s), exposure(s).

Give full and clear descriptions or definitions of the nature of the interventions or the exposures to be reviewed.

Treating teeth loss with endosteal dental implants, undergoing sand-blasting surface modification.

## 21. * Comparator(s)/control.

Where relevant, give details of the alternatives against which the main subject/topic of the review will be compared (e.g. another intervention or a non-exposed control group). The preferred format includes details of both inclusion and exclusion criteria.

Treating teeth loss with endosteal dental implants, with no surface modification (machined surface).

## 22. * Types of study to be included.

Give details of the types of study (study designs) eligible for inclusion in the review. If there are no restrictions on the types of study design eligible for inclusion, or certain study types are excluded, this should be stated. The preferred format includes details of both inclusion and exclusion criteria.

Only randomized controlled trials will be included in the meta-analysis.

## 23. Context.

Give summary details of the setting and other relevant characteristics which help define the inclusion or exclusion criteria.

## 24. * Primary outcome(s).

Give the pre-specified primary (most important) outcomes of the review, including details of how the outcome is defined and measured and when these measurement are made, if these are part of the review inclusion criteria.

Primary outcomes are the number of survived implants at check-ups, and changes in marginal bone level around the implants, which are measured using radiographic images.

## Timing and effect measures

## 25. * Secondary outcome(s).

List the pre-specified secondary (additional) outcomes of the review, with a similar level of detail to that required for primary outcomes. Where there are no secondary outcomes please state ‘None’ or ‘Not applicable’ as appropriate to the review

None

## Timing and effect measures

## 26. Data extraction (selection and coding).

Give the procedure for selecting studies for the review and extracting data, including the number of researchers involved and how discrepancies will be resolved. List the data to be extracted.

## 27. * Risk of bias (quality) assessment.

State whether and how risk of bias will be assessed (including the number of researchers involved and how discrepancies will be resolved), how the quality of individual studies will be assessed, and whether and how this will influence the planned synthesis.

Risk of bias will be assessed according to the Cochrane Handbook, Cochrane Risk of Bias Tool. Included records will be assessed by two review authors independently. Differences between the two reviews will be discussed until agreement is reached. If it is necessary a third review author will be involved. Records which show evidence of no randomization, will be excluded from data synthesis.

## 28. * Strategy for data synthesis.

Give the planned general approach to synthesis, e.g. whether aggregate or individual participant data will be used and whether a quantitative or narrative (descriptive) synthesis is planned. It is acceptable to state that a quantitative synthesis will be used if the included studies are sufficiently homogenous.

In our meta-analysis we will focus on analytical approaches besides descriptive synthesis of the findings. Where appropriate, for continuous data mean difference and for dichotomous data relative risk (RR) values (with 95% confidence interval) will be calculated. In addition, contribution weight of studies and statistical heterogeneity will be also calculated. For statistical significance p 0.05 will be used. Statistical calculations will be performed with STATA software (StataCorp LLC, USA)

## 29. * Analysis of subgroups or subsets.

Give details of any plans for the separate presentation, exploration or analysis of different types of participants (e.g. by age, disease status, ethnicity, socioeconomic status, presence or absence or co-morbidities); different types of intervention (e.g. drug dose, presence or absence of particular components of intervention); different settings (e.g. country, acute or primary care sector, professional or family care); or different types of study (e.g. randomised or non-randomised).

If possible, subgroup analysis will be performed to decrease potential heterogeneity.

## 30. * Type and method of review.

Select the type of review and the review method from the lists below. Select the health area(s) of interest for your review.

### Type of review

Cost effectiveness
No

Diagnostic
No

Epidemiologic
No

Individual patient data (IPD) meta-analysis
No

Intervention
Yes

Meta-analysis
Yes

Methodology
No

Network meta-analysis
No

Pre-clinical
No

Prevention

No

Prognostic
No

Prospective meta-analysis (PMA)
No

Qualitative synthesis
Yes

Review of reviews
No

Service delivery
No

Systematic review
No

Other
No

## Health area of the review

Alcohol/substance misuse/abuse
No

Blood and immune system
No

Cancer
No

Cardiovascular
No

Care of the elderly
No

Child health
No

Complementary therapies
No

Crime and justice
No

Dental
Yes

Digestive system
No

Ear, nose and throat
No

Education
No

Endocrine and metabolic disorders
No

Eye disorders
No

General interest
No

Genetics
No

Health inequalities/health equity

No

Infections and infestations
No

International development
No

Mental health and behavioural conditions
No

Musculoskeletal
No

Neurological
No

Nursing
No

Obstetrics and gynaecology
No

Oral health
Yes

Palliative care
No

Perioperative care
No

Physiotherapy
No

Pregnancy and childbirth
No

Public health (including social determinants of health)
No

Rehabilitation
No

Respiratory disorders
No

Service delivery
No

Skin disorders
No

Social care
No

Surgery
No

Tropical Medicine
No

Urological
No

Wounds, injuries and accidents
No

Violence and abuse
No

## 31. Language.

Select each language individually to add it to the list below, use the bin icon  to remove any added in error.

English

There is not an English language summary

## 32. Country.

Select the country in which the review is being carried out from the drop down list. For multi-national collaborations select all the countries involved.

Hungary

## 33. Other registration details.

Give the name of any organisation where the systematic review title or protocol is registered (such as with The Campbell Collaboration, or The Joanna Briggs Institute) together with any unique identification number assigned. (N.B. Registration details for Cochrane protocols will be automatically entered). If extracted data will be stored and made available through a repository such as the Systematic Review Data Repository (SRDR), details and a link should be included here. If none, leave blank.

## 34. Reference and/or URL for published protocol.

Give the citation and link for the published protocol, if there is one

Give the link to the published protocol.

Alternatively, upload your published protocol to CRD in pdf format. Please note that by doing so you are consenting to the file being made publicly accessible.

No I do not make this file publicly available until the review is complete

Please note that the information required in the PROSPERO registration form must be completed in full even if access to a protocol is given.

## 35. Dissemination plans.

Give brief details of plans for communicating essential messages from the review to the appropriate audiences.

## Do you intend to publish the review on completion?

Yes

## 36. Keywords.

Give words or phrases that best describe the review. Separate keywords with a semicolon or new line. Keywords will help users find the review in the Register (the words do not appear in the public record but are included in searches). Be as specific and precise as possible. Avoid acronyms and abbreviations unless these are in wide use.

## 37. Details of any existing review of the same topic by the same authors.

Give details of earlier versions of the systematic review if an update of an existing review is being registered, including full bibliographic reference if possible.

## 38. * Current review status.

Review status should be updated when the review is completed and when it is published.
Please provide anticipated publication date

Review_Ongoing

## 39. Any additional information.

Provide any other information the review team feel is relevant to the registration of the review.

## 40. Details of final report/publication(s).

This field should be left empty until details of the completed review are available.

Give the link to the published review.
