# Supplementary figures and images for: Sandblasting reduces dental implant failure rate but not marginal bone level loss: A systematic review and meta-analysis
Source: PLoS One. 2019 May 3;14(5):e0216428. doi: 10.1371/journal.pone.0216428 (PMC6499471; doi:10.1371/journal.pone.0216428)

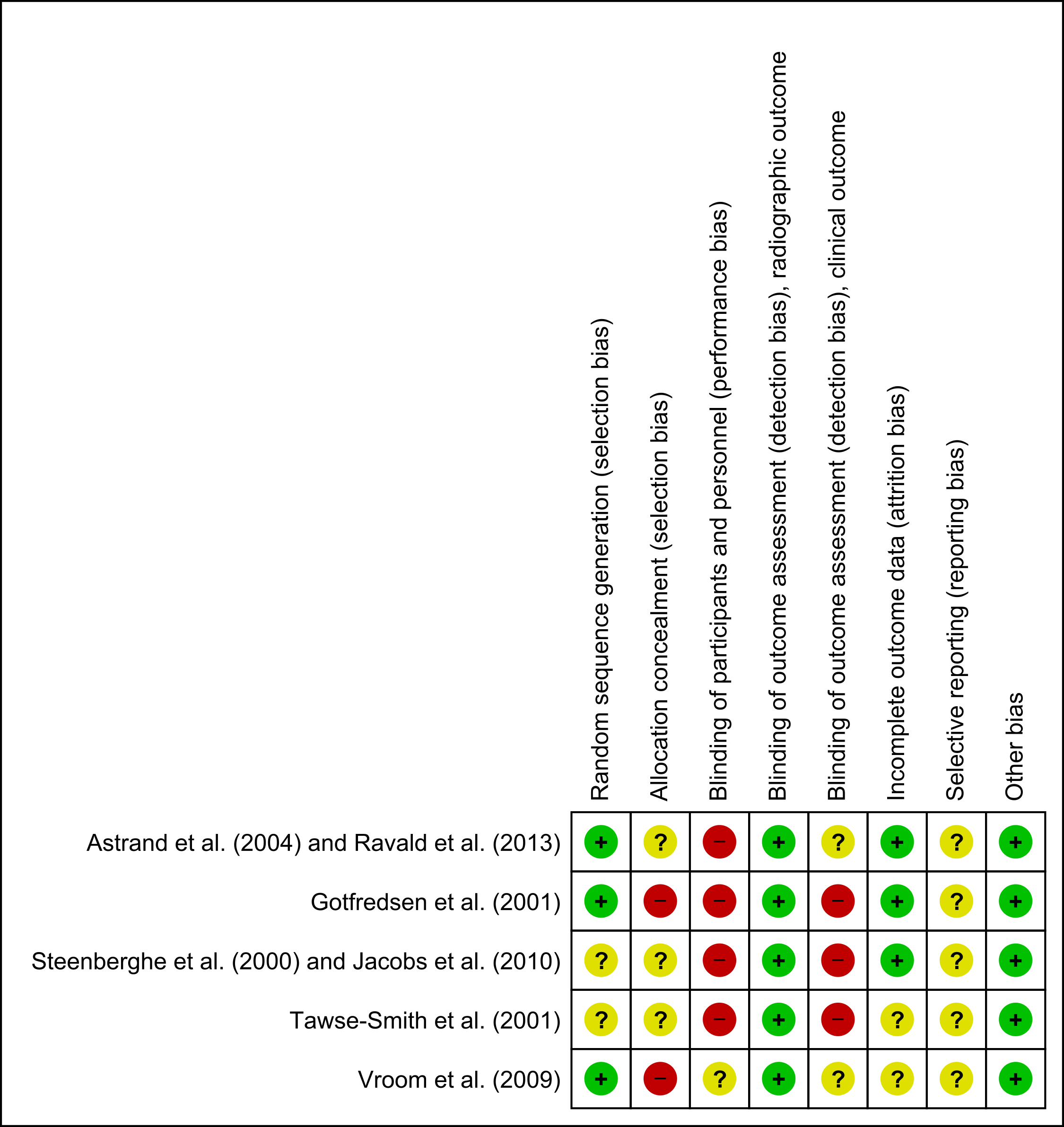

Supplement: S3 Appendix — (TIFF) [file pone.0216428.s003.tiff]
