# Supplementary material for: Sandblasting reduces dental implant failure rate but not marginal bone level loss: A systematic review and meta-analysis
Source: PLoS One. 2019 May 3;14(5):e0216428. doi: 10.1371/journal.pone.0216428 (PMC6499471; doi:10.1371/journal.pone.0216428)

Supplementary Appendix 4. Publication bias (funnel plots).

Funnel plot for pooled risk ratio analysis of implant failure rate after 1 year.

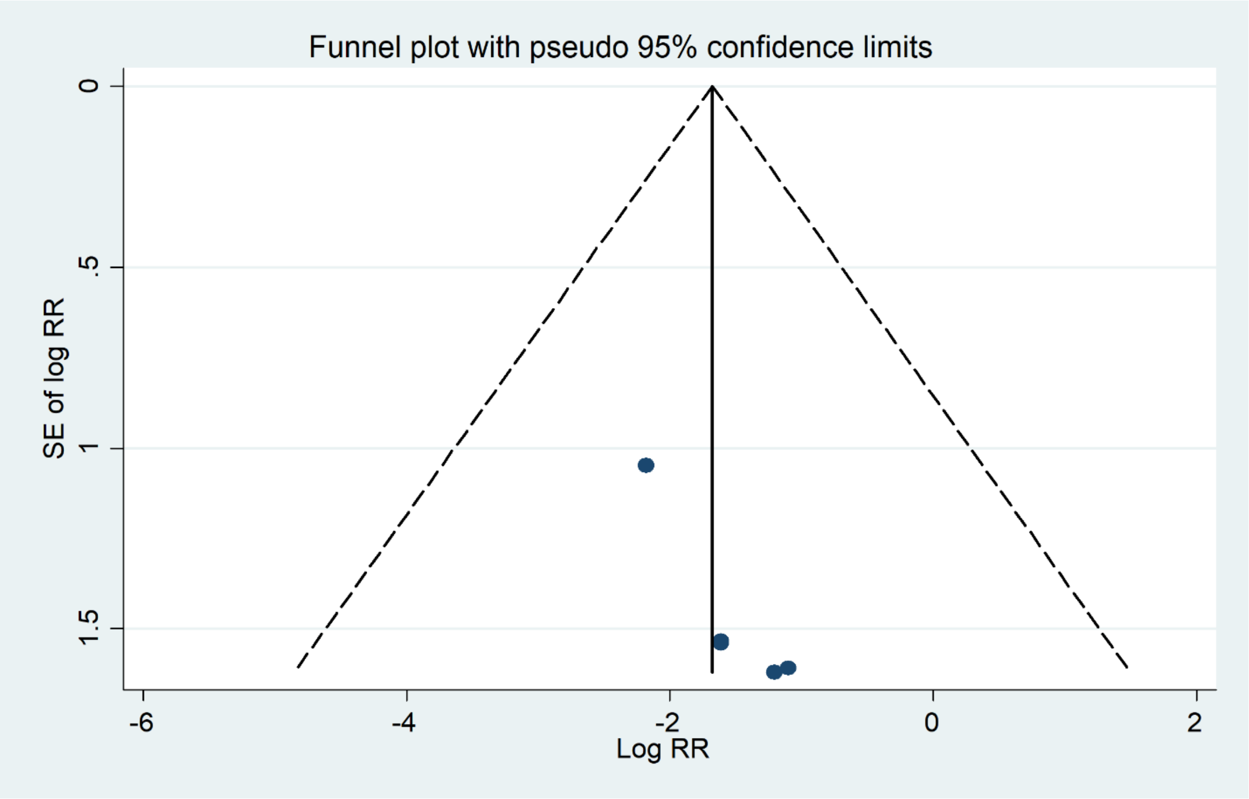

Funnel plot for pooled risk ratio analysis of implant failure rate after 2 years.

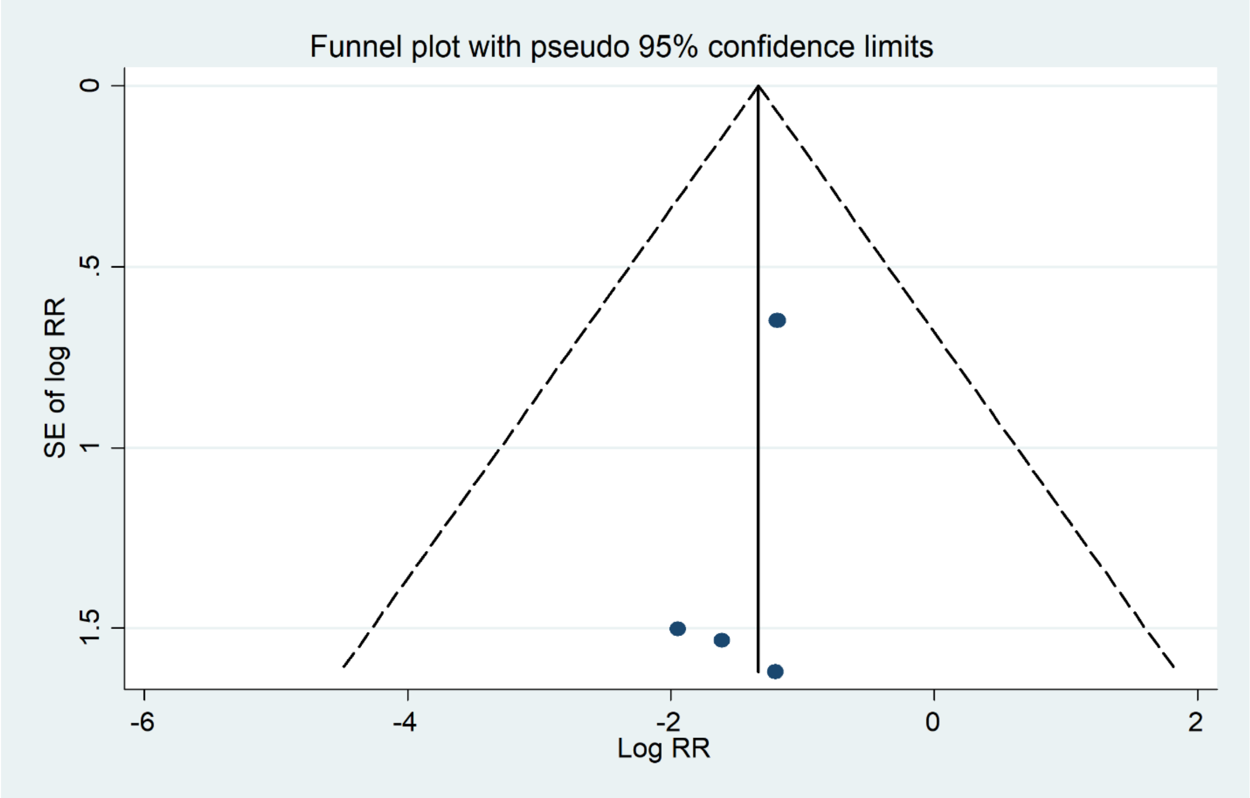

Funnel plot for pooled risk ratio analysis of implant failure rate after 5-6 years.

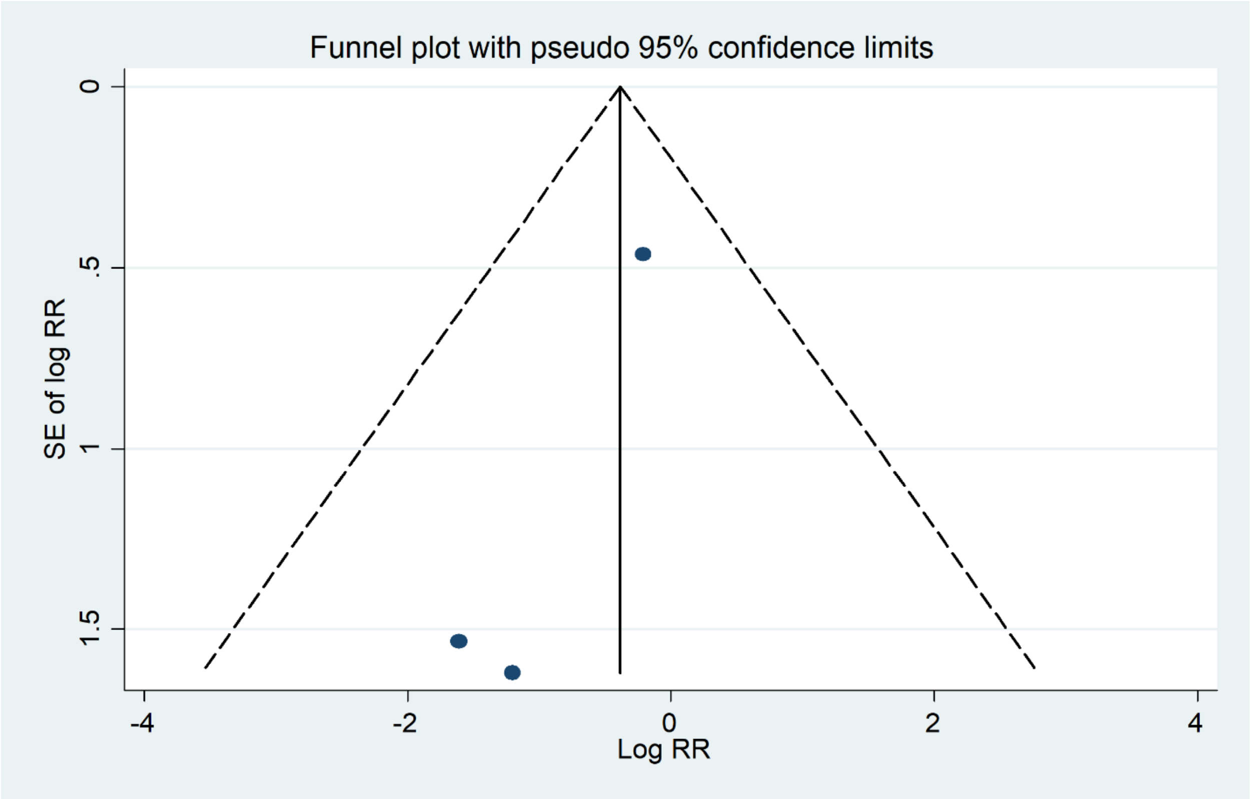

Funnel plot for pooled risk ratio analysis of implant failure rate after 12-15 years.

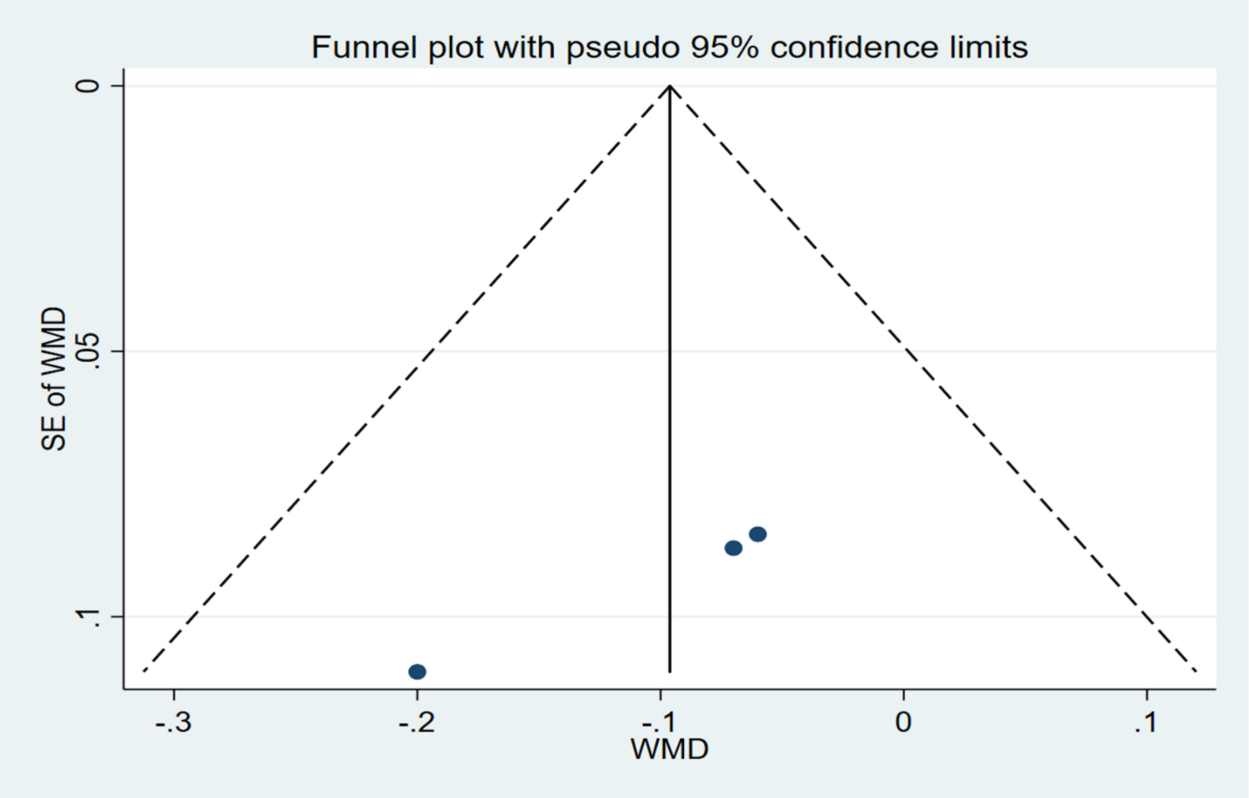

Funnel plot for weighted mean difference of marginal bone level change after 1 year.

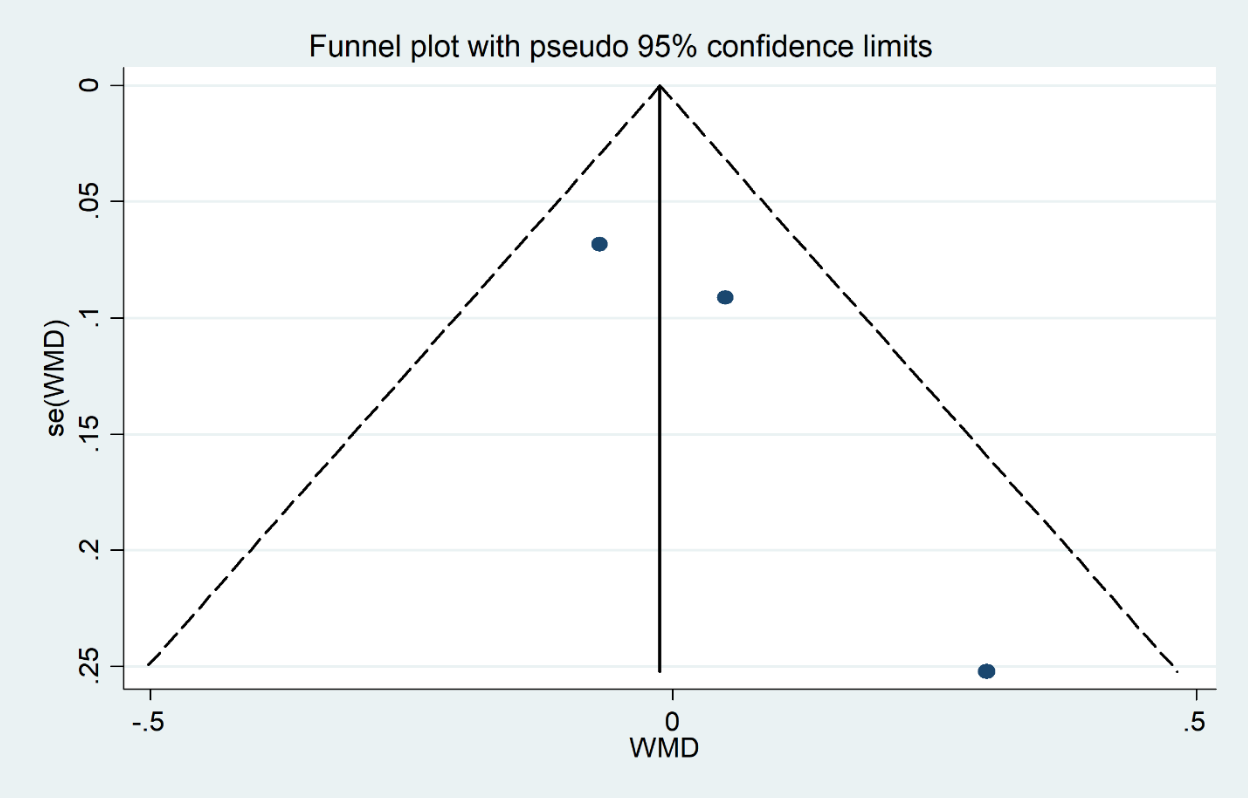

Funnel plot for weighted mean difference of marginal bone level change after 5 years.

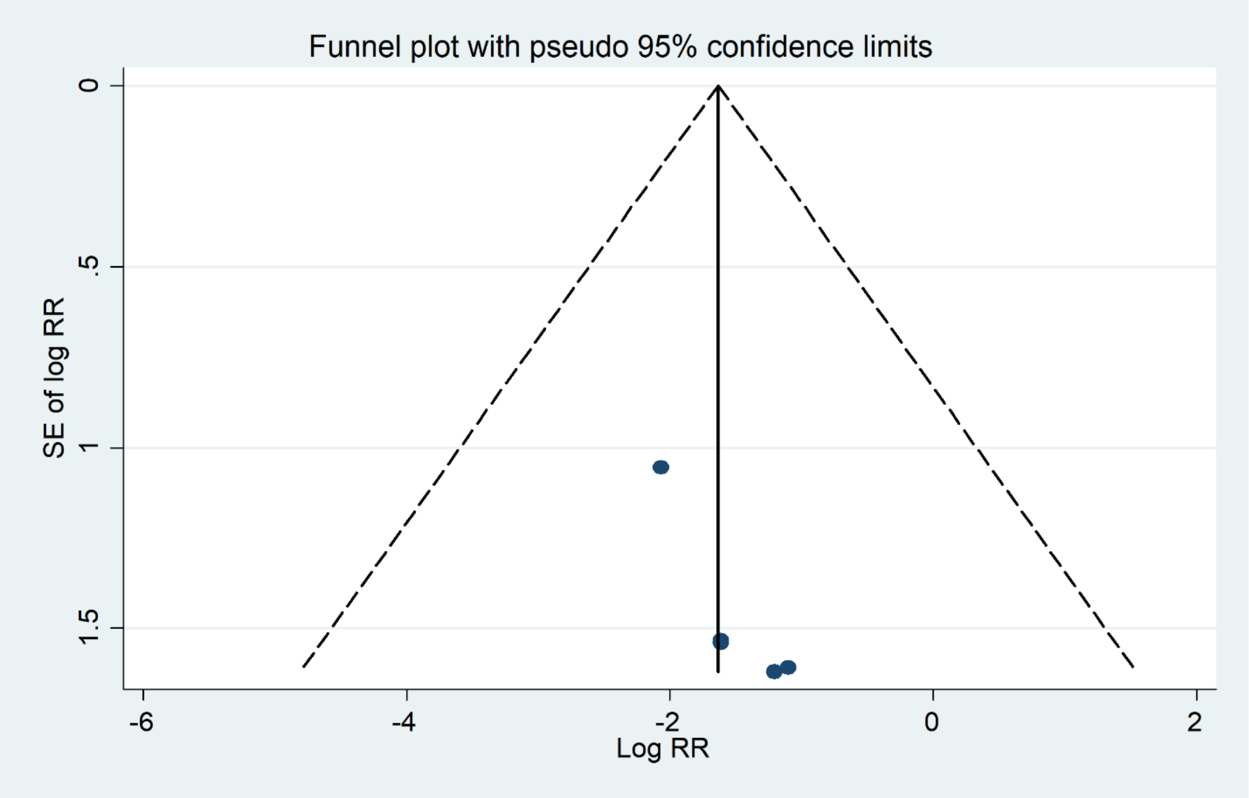

Supplement: S4 Appendix — (PDF) [file pone.0216428.s004.pdf]
