# Supplementary material for: Sandblasting reduces dental implant failure rate but not marginal bone level loss: A systematic review and meta-analysis
Source: PLoS One. 2019 May 3;14(5):e0216428. doi: 10.1371/journal.pone.0216428 (PMC6499471; doi:10.1371/journal.pone.0216428)
Supplement: S1 Table — (PDF) [file pone.0216428.s005.pdf]

**First author and title:** Astrand, P. 2004<br>*Astra Tech and Brånemark system implants: a 5-year prospective study of marginal bone reactions*<br>(The continuation of the study was published by Ravald et al. 2013<br>*Long-term evaluation of Astra Tech and Brånemark implants in patients treated with full-arch bridges. Results after 12-15 years*)		
**Bias**	**Authors judgement**	**Support for judgement**
**Random sequence generation (selection bias)**	Low risk	Quote: "…patients were randomized in blocks with an equal probability of receiving Astra Tech or Brånemark system implants."
**Allocation concealment (selection bias)**	Unclear	Comment: Not described.
**Blinding of participants and personnel (performance bias)**	High risk	Comment: No blinding described, and probably no blinding occurred, due to the fact that different implant systems need different surgical protocols and special abutments for denture fixation thus study personnel must know the implant type.
**Blinding of outcome assessment (detection bias) radiographic outcome**	Low risk	Quote: "A specialist in oral radiology, who did not take part in the clinical treatment, performed the radiographic evaluation."
**Blinding of outcome assessment (detection bias) clinical outcome**	High risk	Comment: Probably no blinding due to the nature of outcome.
**Incomplete outcome data (attrition bias)**	Low risk	Comment: 3 patients were excluded, one lost the implants (Brånemark), the other two died (Astra). However, all patients were included in the cumulative survival analysis.
**Selective reporting (reporting bias)**	Unclear risk	Comment: No access to study protocol or trial registry entry, but no intext evidence of reporting bias.
**Other bias**	Low risk	Comment: Study appears to be free of other sources of risk.

**First author and title:** Gotfredsen, K. 2001<br>*A prospective 5-year study of fixed partial prostheses supported by implants with machined and TiO_2-blasted surface*		
**Bias**	**Authors judgement**	**Support for judgement**
**Random sequence generation (selection bias)**	Low risk	Quote: ”A stratification and a randomization of the 2 surface groups were done. The first implants were selected at random by drawing lots…”
**Allocation concealment (selection bias)**	High risk	Quote: “…thereafter the implants with different surface configurations were inserted alternately.”
**Blinding of participants and personnel (performance bias)**	High risk	Comment: No blinding described, and probably no blinding occurred, due to the fact that different implant systems need different surgical protocols and special abutments for denture fixation thus study personnel must know the implant type.
**Blinding of outcome assessment (detection bias) radiological outcome**	Low risk	Quote: “An experienced radiologist, not otherwise involved in the study, evaluated all the radiographs, blindly.”
**Blinding of outcome assessment (detection bias) clinical outcome**	High risk	Comment: No blinding described, due to the difference in characteristics of the two implant systems evaluators could differentiate between the two.
**Incomplete outcome data (attrition bias)**	Low risk	Comment: 10 % of participants dropped out (eight from both implant types), otherwise no missing data.
**Selective reporting (reporting bias)**	Unclear risk	Comment: No access to study protocol or trial registry entry, but no intext evidence of reporting bias.
**Other bias**	Low risk	Comment: Study appears to be free of other sources of risk.

Article's first author and title: Steenberghe, D 2000<br>*A prospective split-mouth comparative study of two screw-shaped self-tapping pure titanium implant systems*<br>(The continuation of the study was published by Jacobs et al. 2010<br>*A split-mouth comparative study up to 16 years of two screw-shaped titanium implant systems*)		
**Bias**	**Authors judgement**	**Support for judgement**
**Random sequence generation (selection bias)**	Unclear risk	Quote: "…randomized for the jaw in which both implant systems were applied."
**Allocation concealment (selection bias)**	Unclear risk	Comment: Way of randomization and allocation concealment are not described in the study.
**Blinding of participants and personnel (performance bias)**	High risk	Comment: No blinding described, and probably no blinding occurred, due to the fact that different implant systems need different surgical protocols and special abutments for denture fixation thus study personnel must know the implant type.
**Blinding of outcome assessment (detection bias) radiological outcome**	Low risk	Comment: Blinding of outcome assessment not described but unlikely to affect measurement of this outcome.
**Blinding of outcome assessment (detection bias) clinical outcome**	High risk	Comment: No blinding described, due to the difference in characteristics of the two implant systems evaluators could differentiate between the two.
**Incomplete outcome data (attrition bias)**	Low risk	Comment: No drop outs.
**Selective reporting (reporting bias)**	Unclear risk	Comment: No access to study protocol or trial registry entry, but no intext evidence of reporting bias.
**Other bias**	Low risk	Comment: Study appears to be free of other sources of risk.

Article's first author and title: Tawse-Smith, A. 2001<br>*One-stage operative procedure using two different implant systems: a prospective study on implant overdentures in the edentulous mandible*		
**Bias**	**Authors judgement**	**Support for judgement**
**Random sequence generation (selection bias)**	Unclear risk	Quote: "participants were randomly selected from those requesting the placement of osseointegrated Implants…"<br>Comment: However, the method for randomization is not described.
**Allocation concealment (selection bias)**	Unclear risk	Comment: Selection method not described.
**Blinding of participants and personnel (performance bias)**	High risk	Comment: No blinding described, and probably no blinding occurred, due to the fact that different implant systems need different surgical protocols and special abutments for denture fixation thus study personnel must know the implant type.
**Blinding of outcome assessment (detection bias) radiological outcome**	Low risk	Comment: Blinding of outcome assessment not described but unlikely to affect measurement of this outcome, "standardized intraoral radiographs" were used.
**Blinding of outcome assessment (detection bias) clinical outcome**	High risk	Comment: No blinding described, due to the difference in characteristics of the two implant systems evaluators could differentiate between the two.
**Incomplete outcome data (attrition bias)**	Unclear risk	Comment: Two participants dropped out, no reason was given.
**Selective reporting (reporting bias)**	Unclear risk	Comment: No access to study protocol or trial registry entry, but no intext evidence of reporting bias.
**Other bias**	Low risk	Comment: Study appears to be free of other sources of risk.

Article's first author and title: Vroom, M. G. 2009<br>*Effect of surface topography of screw-shaped titanium implants in humans on clinical and radiographic parameters: a 12-year prospective study*		
**Bias**	**Authors judgement**	**Support for judgement**
**Random sequence generation (selection bias)**	Low risk	Quote: "The type of implant surface texture used at this location was randomly assigned using a computer-generated randomization schedule."
**Allocation concealment (selection bias)**	High risk	Quote: "Thereafter, the two types of implants were placed alternatively…"
**Blinding of participants and personnel (performance bias)**	Unclear risk	Comment: The blinding was not described.
**Blinding of outcome assessment (detection bias) radiographic outcome**	Low risk	Comment: Blinding of outcome assessment not described but unlikely to affect measurement of this outcome.<br>Quote: "Standardized intra-oral radiographs were… made… The radiographs … were analyzed using a commercially available dental X-ray software program".
**Blinding of outcome assessment (detection bias) clinical outcome**	Unclear risk	Comment: The blinding was not described.
**Incomplete outcome data (attrition bias)**	Unclear risk	Comment: At the 12 years checkup the sample size decreased by 7. Authors give no explanation.
**Selective reporting (reporting bias)**	Unclear risk	Comment: No access to study protocol or trial registry entry, but no intext evidence of reporting bias.
**Other bias**	Low risk	Comment: Study appears to be free of other sources of risk.
